# Honeycomb-like aluminum antennas for surface-enhanced infrared absorption sensing

**DOI:** 10.1515/nanoph-2023-0131

**Published:** 2023-04-10

**Authors:** Melissa Najem, Franck Carcenac, Luka Coutaud, Mohamed Mouhibi, Thierry Taliercio, Fernando Gonzalez-Posada

**Affiliations:** University of Montpellier, Institut d’Electronique et des Systèmes, Montpellier, Occitanie, France; CNRS Laboratory for Systems Analysis and Architecture, Toulouse, Occitanie, France

**Keywords:** aluminum, honeycomb nanoantennas, MIM, plasmonics, SEIRA spectroscopy, wide tunability

## Abstract

Surface-enhanced infrared absorption (SEIRA) spectroscopy is a competent method to detect trace quantity of molecules and even protein conformational flexibility by enhancing their vibrational modes. To improve the spectroscopy features, we propose a surface with honeycomb-like (HC) arrangement of aluminum equilateral triangles within a metal-insulator-metal configuration. With adjustable geometric parameters, the HC nanoantennas allow a tunable and wide spectral coverage in the IR. The reflectance measurements correlate extremely well with the numerical simulations. Being compact and insensitive to the light polarization, the HC are appealing for boosting the signal-to-noise ratio and increasing the number of hotspots as required for sensing applications. These nanoantennas are thus suitable for accurate and broadband SEIRA sensing via a spectral overlap between the large plasmonic resonances and the narrow IR vibrational modes of our analyte (vanillin). In line with our previously studied bowties nanoantennas, we demonstrate, using HC, SEIRA enhancement factors greater than 10^7^ achieved at a tuning ratio below 1 stating the best spectral overlap. Around 10^4^ molecules are sensed per HC tip. The investigation results are matching the best-reported SEIRA studies. These findings pave the way toward sensitive, adaptable, and miniaturized IR spectroscopy devices for vital applications like biosensing and environmental monitoring.

## 1 Introduction

Customized plasmonic nanostructures are building blocks for various optical applications, among those sensing. Surface-enhanced infrared absorption (SEIRA) is an appealing technique for different domains, such as electrochemistry [1], bioelectrochemistry [2], and sensing [3]. The term “SEIRA” has been first announced by chemists in the early 90s [4]. Since then, it has been massively introduced as a molecule detection and identification method [5]. It capitalizes on the excitation of the localized surface plasmons as collective oscillations of the free-electron cloud of a metallic structure [6]. The molecular fingerprints are identified by resonant detection of their vibrational modes through the coupling to the localized surface plasmons resonance (LSPR) of the nanostructures [7]. Once the LSPR (*ω*_res_) is situated at the same frequency as a molecular vibration (*ω*_vib_), the nanostructure-molecule system can couple [8], resulting in spectral features with Fano-like lineshapes [5, 9], characteristics of a coupling between a broad and a narrow energy states [10]. The Fano-like profiles depend on a tuning ratio between the plasmonic and molecular mode frequencies. Following such coupling, their interaction cross-section increases [5]. Hence, SEIRA overcomes the limitation of conventional IR absorption techniques that cannot afford good sensitivity due to small IR absorption cross-sections such as for gases, or very thin samples as monolayers.

The enhanced IR absorption can only originate from the molecules located within the hotspots of plasmonic nanostructures [5, 11]. Sharp and closely-placed nanoantennas are thus desired to enhance the near electric field (E-field) in-plane of the antennas through both the lightning-rod and the gap effects [12]. The characteristics of metallic nanoantennas are widely studied in the literature, like cross antennas [13] and fan-shaped antennas by Brown et al. [14], nanogapped BT antennas by Dong et al. [15], and dendritic resonators by Wallace et al. [16], to name a few. Furthermore, the size of the nanoantennas is a promising degree of freedom to tune their LSPR frequency which goes along with the near E-field localization and enhancement [17]. Based on this, a barcode-like wide spectral coverage can be foreseen across targeted frequency ranges of the electromagnetic spectrum [18, 19]. In addition, a compact and symmetric arrangement could be a smart engineering to ameliorate the plasmonic nanoantennas behavior. Inspired by the most efficient manner of filling space as studied in crystallography [20], equilateral triangles will be arranged in a hexagonal packing that mimics the natural honeycombs. This special organization will allow a symmetry invariance that might be convenient to ease the incident light implementation as well as to improve the signal-to-noise ratio during the measurements.

In this article, we investigate the plasmonic behavior of equilateral triangles organized as honeycomb-like (HC) arrays. These plasmonic nanoantennas are fabricated with aluminum (Al). Lately, Al has demonstrated great promises as alternative material to noble metals, being affordable, abundant, and compatible with silicon technologies [21, 22]. Herein, the HC antennas are incorporated within a metal–insulator–metal (MIM) configuration. The MIM allows a strong lateral confinement of the E-field in the insulator (dielectric) [23, 24]. The combination of both the lateral and the in-plane E-field confinements leads to maximize the E-field intensity in the gap region between the neighboring triangles as required for SEIRA application. LSPR positions of the HC nanoantennas are extended over a broad IR spectral range by controlling the side length of the triangles. This platform is thus capable of enhancing multiple IR vibrational features offering a broadband SEIRA sensing.

Recently, Aslan et al. have employed honeycomb-shaped plasmonic nanoantennas as photonic metamaterials within a MIM structure to demonstrate a potential SEIRA sensor through the detection of a polymer nanolayer [25]. A large electromagnetic near-field enhancement was provided at the activated hotspots that are located at the sharp extremities of a completely-filled HC building-block. However, in our present article, an advanced highly-compact and highly-activated structure is promoted: more hotspots are triggered by filling the HC unit-cell with six sharp equilateral triangles. HC arrays with different sizes are studied experimentally by single detector Fourier transform infrared (FTIR) measurements and by finite-difference time-domain (FDTD) simulations. Their corresponding LSPR are defined in a wide IR range. The invariance of the HC structure to the light polarization is proved. The advantage of the compact HC arrangement is also underlined and supported by a brief comparison to our formerly published bowties (BT) results [18]. SEIRA results are as well compared for both nanoantennas. As a case study, we demonstrate a broadband SEIRA using vanillin as a trial analyte. The vanillin molecule exhibits several sharp modes characteristics of the vibrational analysis of its aromatic ring. The analysis of the resulting Fano-like vibrational signals of a µ-droplet of vanillin/IPA solution shows a maximum enhancement factor higher than 10^7^ reached at a frequency tuning ratio below 1. The number of sensed molecules was analytically found around 10^4^ molecules per HC tip. These results are in agreement with the stated results in the literature as well as in our earlier publication about Al-BT [18]. These outcomes are encouraging for the conception of a compact, cost-effective, and sensitive biosensor based on silicon technologies.

## 2 Methods

### 2.1 Simulation

The electromagnetic response of the HC antenna integrated in a MIM configuration has been simulated by three-dimensional FDTD calculations using Ansys Lumerical 2021 R2.3 Finite difference solver.

The MIM structure has been modeled as a 50 nm-thick aluminum (Al) HC with variable side length *L* and gap *g*, on top of a 100 nm thick Al layer (mirror), with a 20 nm thick dielectric (SiO_2_) layer stacked in between. For the Al, we referred to the dielectric constants reported in CRC Handbook of Chemistry and Physics by E. Shiles [26]. The SiO_2_ material data are based on the Handbook of Optical Constants of Solids I – III by E. Palik [27], but are slightly modified to match the real quality of the deposited material. An axiomatic refractive index equal to 1 (representing air) is maintained in the structure surroundings to create an asymmetric environment.

HC-like pattern is obtained by creating three groups of periodic lines rotated from the horizontal *x*-axis with an angle of 0°, 60°, and 120° degrees within the 50 nm thick Al layer. The lines are defined as etched material. The lines width *w* and periodicity *p* are affecting the sculpted Al triangles size (triangle’s height equal to *L* × sin 60°) and their spacing distance (that is half of the eventual tip-to-tip gap *g*). Symmetric and anti-symmetric boundaries are employed along the *x*- and *y*-direction, and perfect matching layers (PMLs) are along the *z*-direction. A mesh grid of 5 nm is used around the intersection gap. An incident plane-wave is co-or cross-polarized (E-filed component along or across the tip-to-tip facing pair of triangles, respectively) and launched backward along the *z*-axis for illumination. Thereafter, optical responses of Al-HC are collected using a monitor placed above the excitation source, so-called reflectance monitor. A field monitor is introduced in the *xy*-plane to represent the electric field cartography. Throughout both monitors, the influence of geometrical parameters on the plasmonic behavior is studied.

### 2.2 Fabrication

The main substrate fabrication is detailed in our previous work [18]. In addition, onto the 20 nm-thick SiO_2_ layer, a 50 nm-thick Al layer is thermally evaporated at 1 Å/s.

On top of this layer, the HC patterns are engineered by electron beam lithography (EBL) using dilute positive tone resist (CSAR) within the extent of 100 µm × 100 µm. A Schottky-emitter field emission gun (FEG) EBL system (RAITH150 equipment) is used with a 10 µm aperture, operating at 20 kV (beam current of 40 pA), a working distance of 7 mm. An area stepsize of 4 nm with an area nominal dose of 50 μC/cm^2^; and a line stepsize of 2 nm with a line nominal dose of 100 pC/cm^2^ are selected. During the exposure, the electron beam is intentionally scanned over the sample to define a matrix of 5 × 11 zones, in each of which *L* and *g* are fixed. After electron exposure, the exposed resist is developed in an amyl acetate solution for 60 s, and then the sample is immediately rinsed in IPA (stopper solution) for 30 s.

To transfer the patterns into the metal layer, the 50 nm-thick Al layer is etched via the opened lines using argon (Ar) sputtering into an ICP-Corial machine (150 W as RF and 200 W as LF, with a voltage bias of 170 V) for 180 s. At the end, the unexposed resist was cleaned by an inductive plasma O_2_ (100 % O_2_) for 5 min.

### 2.3 Characterization

The etching depth is surveyed using atomic force microscopy (AFM) analysis to calibrate the etching rate. The morphology of the tailored HC antennas is examined by a Hitachi S4800 scanning electron microscope (SEM) at 10 keV. The optical response of the fabricated resonators is characterized through a Hyperion 3000 microscope coupled to a Fourier transform IR (FTIR) spectrometer (Bruker Vertex 70). A broadband IR light source, namely Globar (silicon carbide SiC-based-MIR source) with potassium bromide (KBr) beam splitter is implemented for the measurements. The light is focused on the sample through a 36× Cassegrain objective (numerical aperture N.A. = 0.5) and collected onto a liquid N_2_–cooled mercury cadmium telluride detector (MCT or Hg:Cd:Te) with a single element detector. The accessible spectral band is ranging between 400 and 8500 cm^−1^. The acquired spectra are background-corrected to the spectrum of a gold mirror serving as background. Background and spectral measurements are systematically executed using single element configuration with a resolution of 4 cm^−1^, and interferograms from 1000 scans are typically averaged to obtain one background-corrected spectrum, achieving a high signal-to-noise ratio. Co- and cross-polarized illuminations are subsequently introduced as 0° and 90° angles are respectively read on the polarizer goniometer. Unpolarized light is also implemented by removing the polarizer from the optical pathway.

## 3 Results and discussion

**Figure 1:**
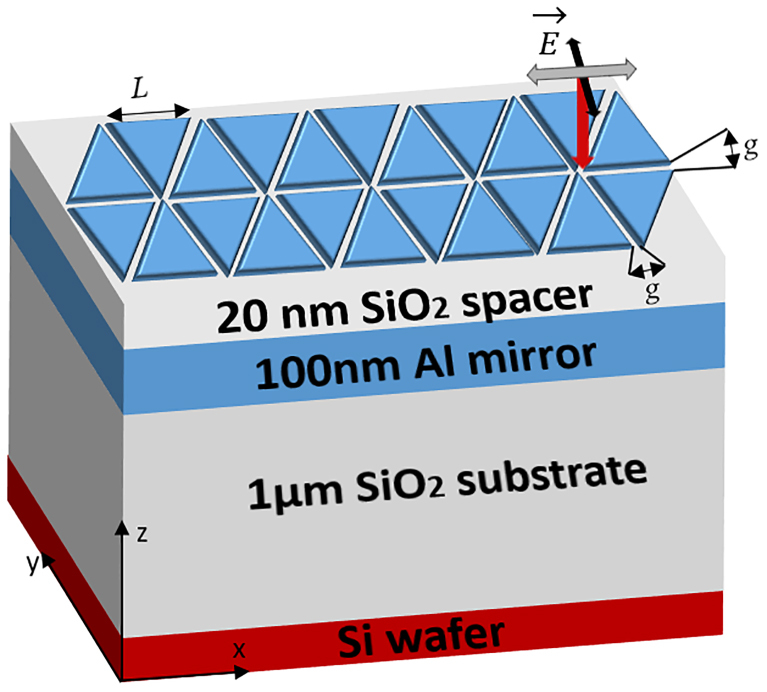
Schematic of the HC nanoantenna arrays integrated in a MIM structure (Al-HC [50 nm]/SiO_2_-spacer [20 nm]/Al-mirror [100 nm]), stacked on a MOS-compatible substrate (SiO_2_/Si). *L* is the equilateral triangle side length; *g* is the tip-to-tip gap. Each HC is considered as an FDTD unit cell and the periodicity is defined by the boundary conditions. Red, black, and grey arrows illustrate, respectively, the directions of the wavevector of the incident-light, the co-polarized electric field, and the magnetic field.

Figure 1 illustrates the MIM structure and the HC array design consisting of equilateral triangles. All the geometrical parameters are denoted in the caption of Figure 1.

A 100 nm mirror layer is sufficiently thick to suppress the light transmission in the IR spectral range. The SiO_2_ insulator thickness was determined by FDTD calculations to maximize the E-field enhancement in agreement with previous studies of Al and Au BT within a MIM configuration, investigated by Wang et al. [28] and Lin et al. [23], respectively.

### 3.1 Nanoengineering

**Figure 2:**
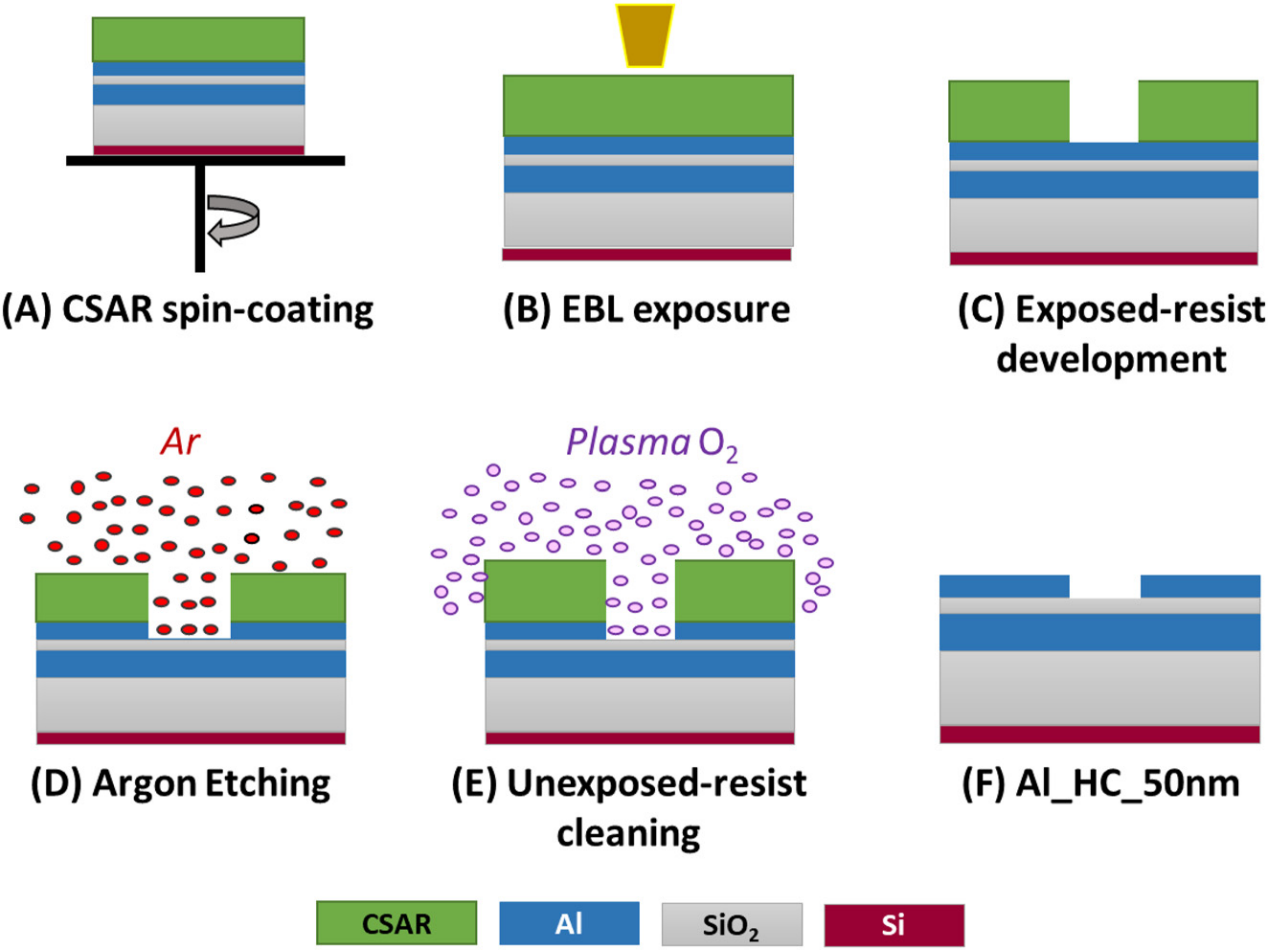
Technological process for HC starts from (A) CSAR resist spin-coating, followed by (B) an EBL exposure and (C) a standard development, then (D) an argon etching and ending with (E) a resist pure plasma O_2_ cleaning process, to retrieve (F) the final Al-HC.

The HC antennas are fabricated by combing an EBL and a dry Ar etching procedure. By this strategy, HC nanoantennas with controlled *L* and *g* are created consisting of hexagonally-arranged equilateral triangles. Overall, *L* values are varied from 2.0 to 1.0 µm with a step of 0.1 µm; and *g* from 0.1 to 0.02 µm with a step of 0.02 µm. The technological process flow is summarized in Figure 2 as the following: (A) positive tone (CSAR) resist spin-coating on top of the 50 nm-thick Al layer, (B) EBL linear exposure, (C) exposed-resist development, (D) metallic dry etching by argon (Ar) sputtering for 180 s, (E) unexposed-resist cleaning in a 100 % plasma O_2_ environment, and (F) the final structure of 50 nm-thick Al-HC arrangement.

**Figure 3:**
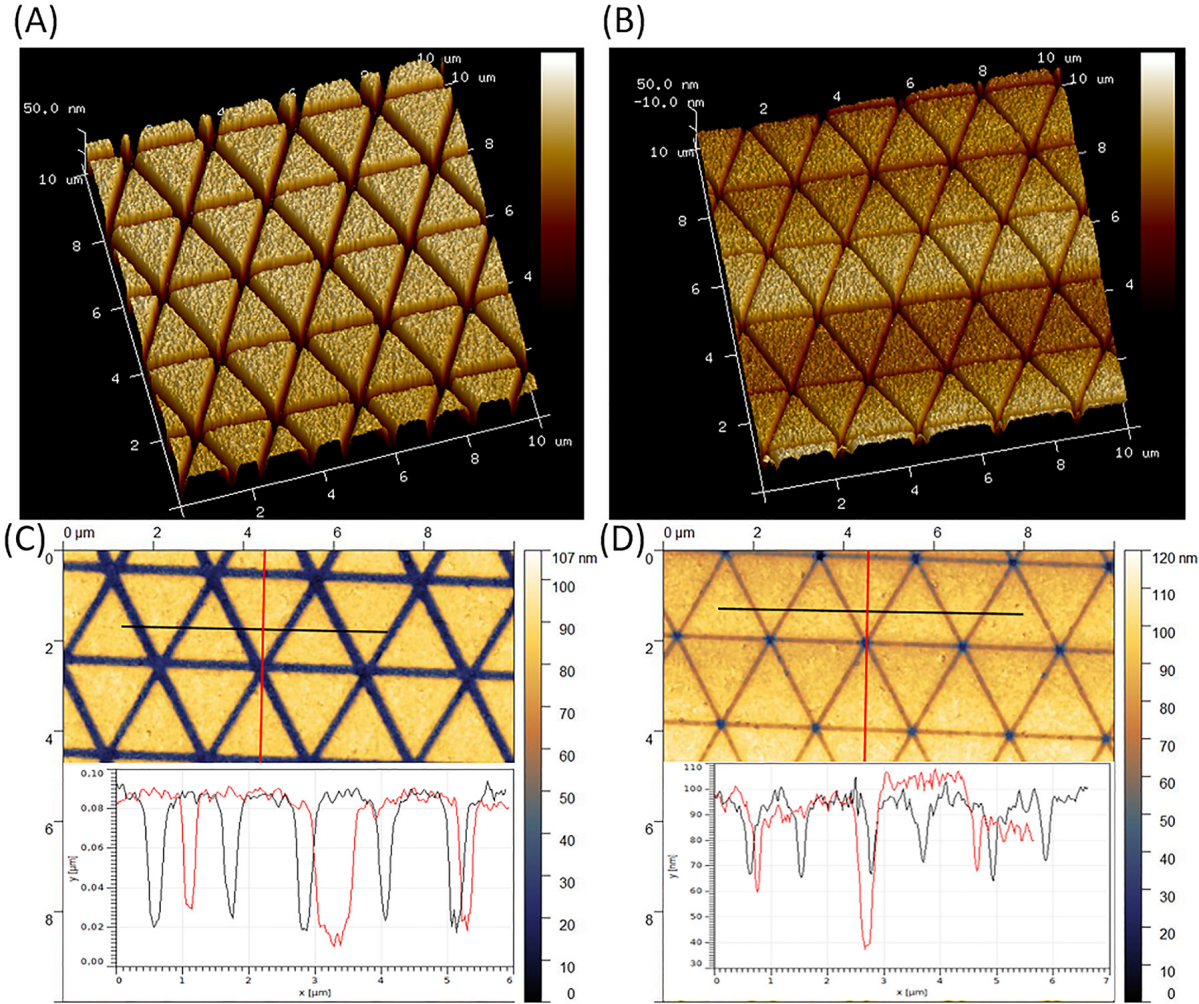
AFM images at the end of the process for *L* = 2.0 µm and *g* equal to (A, C) 0.1 and (B, D) 0.02 µm. Ar etching time is set to 180 s. Color scale intensity is varied between 50 and 70 in (A) and (B) for better visualization. The surface of the Al triangles is normalized likewise in both cases, and the lines definition in (A) is stating a very clean and directional etch. The depth in (C) is standardized around 55 ± 2 nm, but in (D) a deeper etch occurs at the intersection spots, and the lines are not perfectly open.

The etching rate is proportional to the opening width that is used to transfer the patterns towards the metallic layer. Hence, the etching step is challenging to optimize as all the *g* values (line width) are engaged on the same sample surface. Nevertheless, to prove that the desired depth of 50 nm is achieved with Ar etching, AFM measurements are done as detailed in Figure 3 for *L* = 2.0 µm and opening of 0.1 and 0.02 µm. Figure 3A underlines the verticality of the triangle’s profiles following the etching step by Ar sputtering. Based on Figure 3C and D, the RMS values of the triangle’s surface are alike for both cases and respectively equal 2.5 ± 0.3 nm and 2.6 ± 0.7 nm. To evaluate the etching depth, two cross-sections are sketched in Figure 3C and D along (in red) and across (in black) the tip-to-tip facing triangles. In Figure 3C, a global depth value is found around 55 ± 2 nm through the in-between lines, and a slightly deeper value is found at the intersection point. However, for a smaller opening (Figure 3D), the expected depth is only found at the intersection point. In principle, at the intersection of the exposed lines, the width of the opening is wider than that of the lines between the edges of the triangles. This is due to the triply exposed resist at the intersection sites. Except for these spots, the depth is found around 27 ± 2 nm. Hence, it would have been better to separate the large and the small opening on two different samples and to etch the small openings for a longer time.

**Figure 4:**
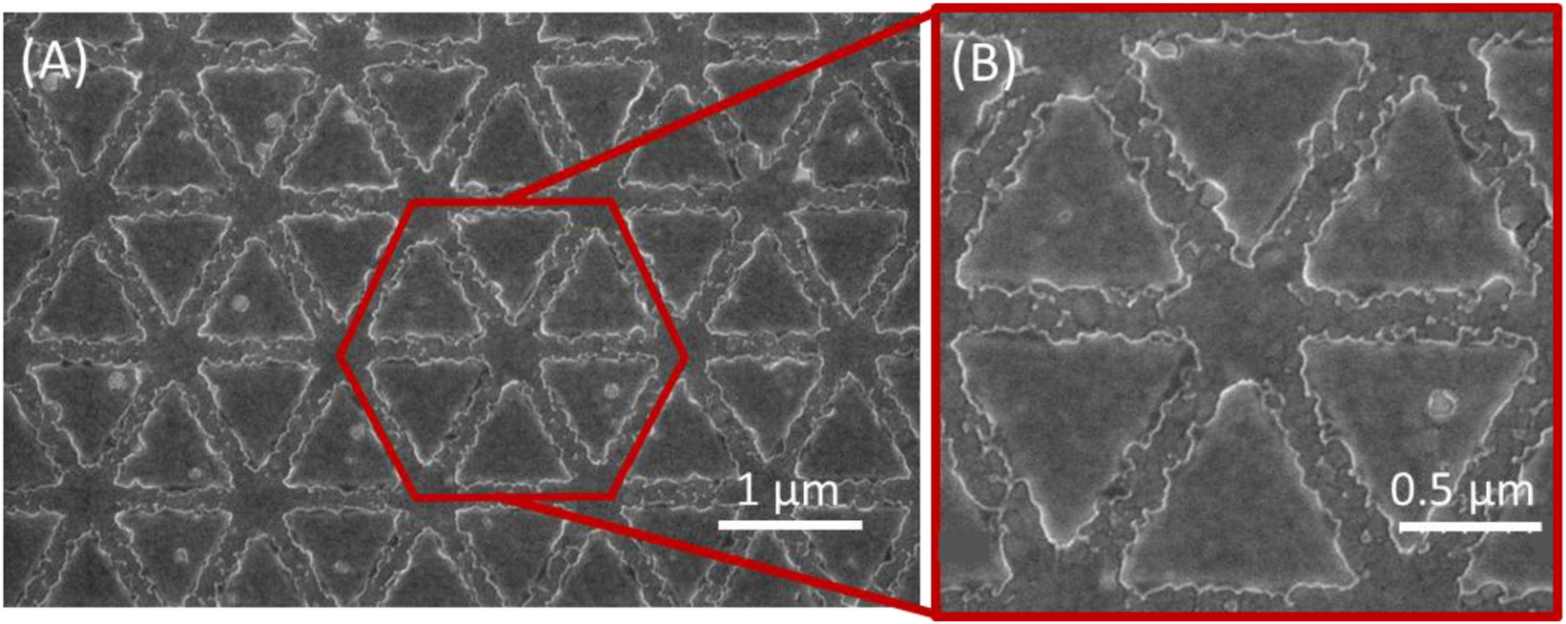
SEM images of the HC patterns. *L* = 1.0 µm and *g* = 0.1 µm in both (A, B), but the magnification is changed from ×20.0 k (A) to ×40.0 k (B). The triangle finishing is damaged during the Ar etching due to the presence of oxide grains on the sample surface.

Complementary to the AFM images, those collected by SEM highlight the effect of the oxide grains on the final shape of the transferred triangles. As revealed in the SEM images (Figure 4A and B) for *L* = 1.0 µm and *g* = 0.1 µm, a damaging etch occurs due to the oxide grain’s resistance against the Ar bombardment. Their resistance is redirecting the Ar ions towards the side walls of the Al triangles. Thus, it is recommended to improve the smoothness of the multi-stacked layers.

Although the triangle’s sharpness is not impeccably reached, these HC antennas are robust and fairly adequate to retrieve a good optical behavior compared to the simulation and to successfully cover a wide IR range as proved in the upcoming sections.

### 3.2 Optical characterization

#### 3.2.1 Wide spectral coverage

**Figure 5:**
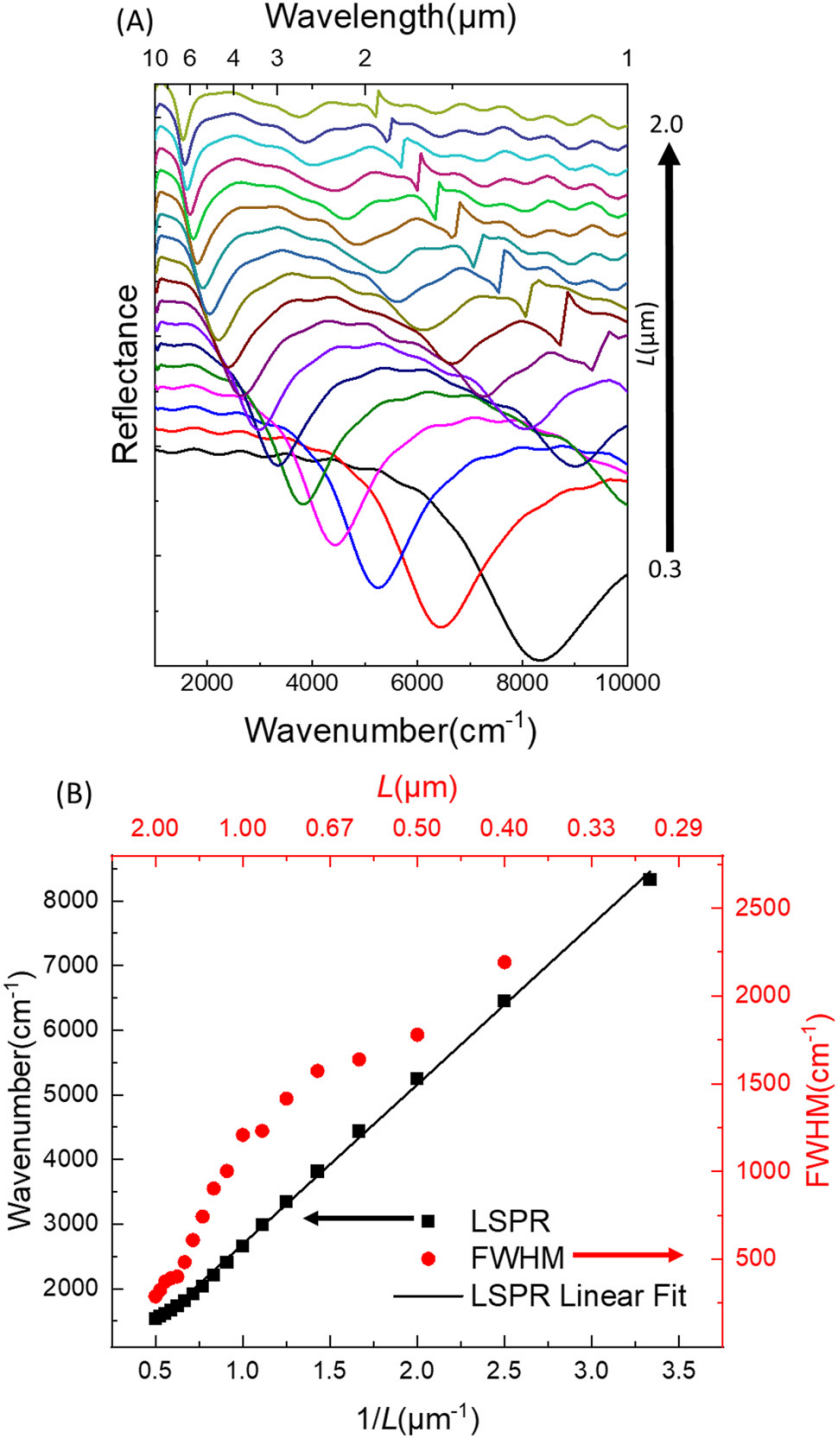
Influence of *L* value on the LSPR spectral position. (A) Numerical (FDTD) reflectance results for HC illuminated under co-polarized light, where *g* is kept constant at 100 nm and *L* is varied from 0.3 to 2 µm with a step of 0.1 µm. Note the spectra are stacked along the reflectance axis for ease of viewing. (B) LSPR wavenumber values are plotted in function of 1/*L* and fitted by a linear fit (black line), and their corresponding width (FWHM) is showing smaller values as the *L* increases. The black arrow refers to the LSPR wavenumbers scale (to the left). The red arrow refers to the FWHM values scale (to the right).

Al-HC is illuminated under a co-polarized (co-pol) plane-wave light source, and the E-field is parallel to the vertical axis of the tip-to-tip facing triangles (black arrow in Figure 1). The reflectance spectra of various Al-HC are simultaneously collected through an *xy*-plane-monitor placed above the incident source level. Similar to their Al-BT counterparts, the Al-HC are able to cover a wide IR spectral range thanks to their tunable LSPR. Hence, a wide spectral coverage from NIR to MIR is insured using Al-HC from 1000 to 10,000 cm^−1^ as plotted in Figure 5A. In particular, the LSPR of the HC cover the range between 1.0 and 6.6** **µm (*i.e.* 10,000 to 1540** **cm^−1^ with a step of ∼70 ± 30 cm^−1^), by varying *L* from 0.3 to 2.0 µm with a step of 0.1 µm. For all HC, the first-order of LSPR is always established at the lowest wavenumber (*i.e.* lowest energy), and then higher orders of the resonance appear at higher wavenumbers (Figure 5A). This behavior corresponds to the dipolar mode which demonstrates a wavelength shift as *L* varies. This well-known tendency for metallic objects is generally defined in the literature [17, 29, 30]. Some higher-order radiative modes are visible only for *L* > 0.7 µm with a similar tendency. The spectral position of the diffraction peaks Λ_*y*_ are resulting from their periodic arrangement which is triggered herein along the *y*-axis and Λ_*y*_ has been analytically deduced in Equation (1) as it follows:

(1) $$\Lambda_{y}=L\times \sin60{}^{\circ}+\sqrt{3}\times g.$$

Considering the behavior of the LSPR wavelength (*λ*_res_) to red-shift as soon as *L* increases (Figure 5A), the LSPR wavenumbers *ω*_res_ (as proportional to 1/*λ*_res_) are plotted as a function of 1/*L* in Figure 5B. In analogy to the behavior of a vibrating string, the anticipated linear dependency is confirmed by the automatic linear fit with the coefficient of determination *R* ^2^ = 0.99 and a slope coefficient of 0.2470. Though, as a multiresonant MIM system is hosted, one should discuss the properties of such a plasmonic waveguide. To get more physical insight, the MIM resonator is analyzed as a Fabry–Perot cavity. The thickness of the dielectric (insulator) layer, denoted as *t*_*d*_, is a key factor in MIM cavities. For a *t*_*d*_ >>> *λ*, the waveguide is formed by uncoupled surface plasmons (SPs) propagating at the two semi-infinite metal/dielectric interfaces. However, as soon as *t*_*d*_ decreases (*t*_*d*_ <<< *λ*), the SPs propagating at both interfaces are coupled, *i.e.* these modes interact more strongly, thus, the effective index *n*_eff_ in this narrow cavity rapidly increases [31]. The effective index for the fundamental waveguide mode is given by Equation (2) as:

(2) $$n_{\text{eff}}^{t_{d}<<<\lambda }=\varepsilon_{d}^{1\;/2}{\left(1-\frac{i\lambda }{\pi t_{d}\varepsilon_{m}^{1/2}}\sqrt{1-\frac{\varepsilon_{d}}{\varepsilon_{m}}}\right)}^{2}$$

For such a subwavelength cavity the effective index of reflection will be written as $n_{\text{eff}}={\tilde{n}}_{\text{eff}}+i{\tilde{k}}_{\text{eff}}$ , and with good approximation, ${\tilde{n}}_{\text{eff}}$ = $\varepsilon_{d}^{1\;/2}$ = 1.3 (SiO_2_ as dielectric).

As one of the metallic layers has a finite length (*L*) and based on the Fabry–Perot phase matching condition, ${\tilde{n}}_{\text{eff}}$ is expressed at the resonance wavelength (*λ*_res_) as in Equation (3):

(3) $${\tilde{n}}_{\text{eff}}=\frac{\lambda_{\text{res}}}{2}\frac{1}{L}\left(m-\frac{\phi_{r}}{\pi }\right)$$

in which, *m* is an integer equal to 1, 2, … representing the mode number, and *ϕ*_*r*_ is the phase of the modal reflection coefficient at the air/MIM interface by having *r* = $\sqrt{R}{\text{e}}^{\text{i}\phi_{r}}$ . The reflection phase *ϕ*_*r*_ varies depending on the cavity dimensions. As detailed in Equation (3), the coefficient of proportionality between 1/*λ*_res_ and 1/*L* depends on ${\tilde{n}}_{\text{eff}}$ , *m* and *ϕ*_*r*_ as $\frac{1}{\lambda_{\text{res}}}=\frac{\left(m-\frac{\phi_{r}}{\pi }\right)}{2{\tilde{n}}_{\text{eff}}}\frac{1}{L}$ . For the fundamental resonance mode (*m* = 1), and by recalling the value of the slope coefficient calculated in Figure 5B, the reflection phase *ϕ*_*r*_ of our multiresonant MIM system is around $\frac{\pi }{3}$ .

Furthermore, the simulated FWHM (full width at half maximum of the resonance) are revealing a decrease while going deeper in the IR, *i.e.* for bigger HC antennas (with larger *L*). The LSPR is evaluated as well through a *Q-*factor expressed in Equation (4) as:

(4) $$Q\text{-}factor=\lambda_{\text{res}}/FWHM$$

where, *λ*_res_ is the central wavelength of the LSPR.

For large HC, 1.4 µm < *L* < 2.0 µm, the *Q-*factor mean value is 4.2 ± 0.7. It is higher than the one found for smaller NA for 0.3** **µm < *L* < 1.0 µm (FWHM mean value is 2.5 ± 0.2). This reduction of the *Q-*factor is most likely affected by the Al internal losses (interband transition mode) situated between *λ* = 0.8 and 0.9 µm (fairly close to 10,000 cm^−1^). Al losses are due not only to interband transitions but also to Drude scattering. In both cases, the corresponding *Q-*factors are considered in the same order of magnitude.

**Figure 6:**
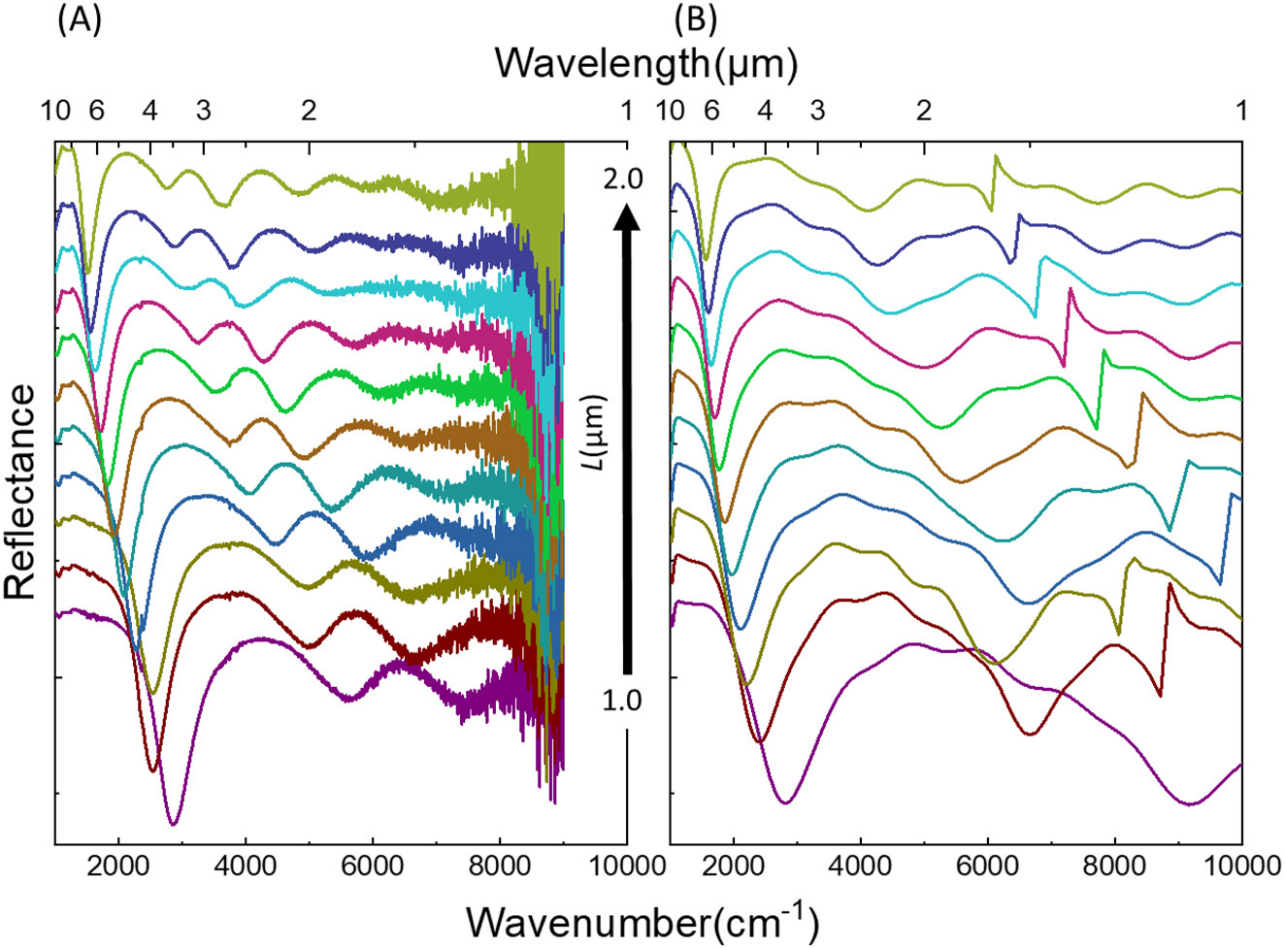
Plasmonic activation of the optical transducer over a broad spectral band in the IR according to the side length of the hexagonally placed equilateral triangles in each HC array zone. (A) Experimental (FTIR) versus (B) numerical (FDTD) reflectance results for HC arrays illuminated under co-polarized light (E-field parallel to the axis of the tip-to-tip facing triangles), where *g* is kept constant at 100 nm and *L* is varied from 1.0 to 2.0 μm with a step of 0.1 μm. Note the spectra are stacked along the reflectance axis for ease of viewing.

The influence of *L* on the LSPR is also observed using the FTIR under a co-polarized (co-pol) light. Here *L* is changed from 1.0 and 2.0 µm with a step of 0.1** **µm. The corresponding LSPR reflectance spectra are plotted in Figure 6. Figure 6A and B show respectively the measured (FTIR) and the simulated reflectance spectra (FDTD). Experimentally, as *L* increases, the energy of the main LSPR peaks is progressively reduced from 2800 to 1500 cm^−1^ (red-shifting from 3.57 to 6.6 µm). In particular, while changing the *L* with a step of 0.1 µm, the LSPR is red-shifted by ∼100 ± 50 cm^−1^. A good agreement is deduced by comparing the measured and the simulated LSPR. Though, a slight spectral shift of ∼60 ± 20 cm^−1^ is separating the FTIR-resonance positions and the ones retrieved by FDTD. This shift is mainly due to the deposited material quality.

Despite their similitude, the spiky diffraction peaks that we can observe in the simulation spectra, Λ_*y*_, are suppressed under the FTIR detector having a wide N.A. objective in the microscope. Moreover, in Figure 6A the tangible belongings of the nearby environment, *e.g.* absorption lines of water and CO_2_, are witnessed since the optical measurements are acquired in an N_2_ medium and not in vacuum. The signature of weak oscillations spanning between 1090 and 1800 cm^−1^, 3500 and 3900 cm^−1^, and above 5000 cm^−1^ corresponds to water lines. The ro-vibrational modes of CO_2_ are determined around 2350 cm^−1^, and barely envisaged unless it is perfectly intersecting with the resonance of *L* = 1.3 µm situated at 2283 cm^−1^.

#### 3.2.2 Polarization effect on the LSPR

**Figure 7:**
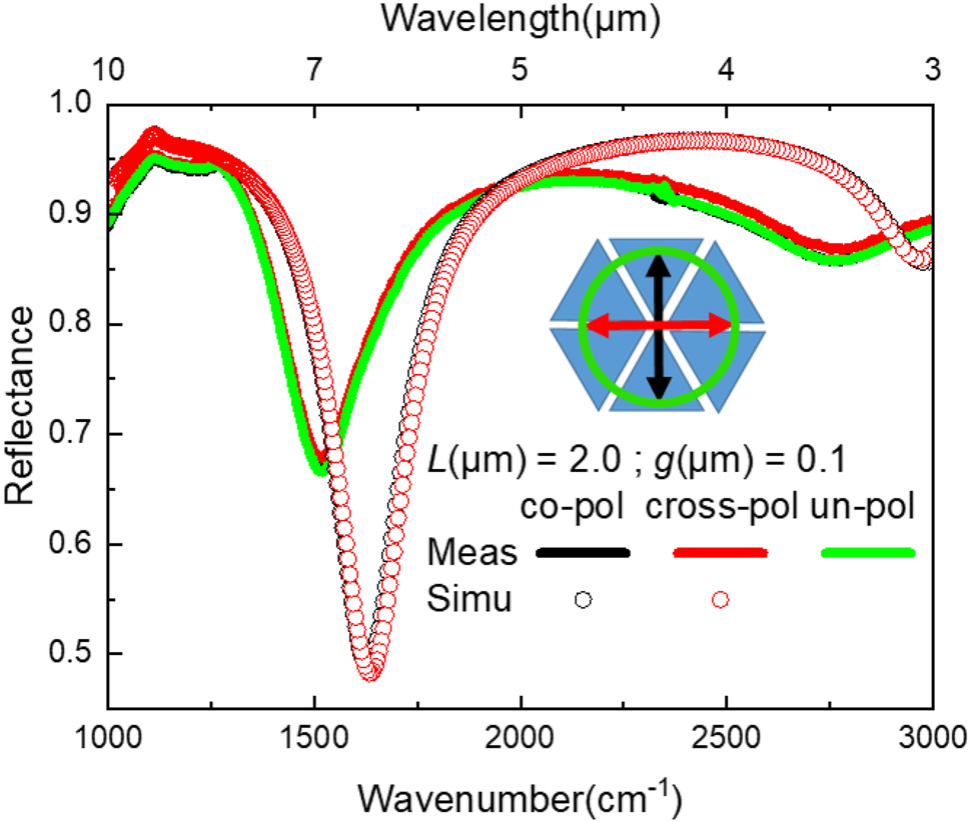
Polarization influence on the LSPR of the HC NA illuminated under co-pol, cross-pol, and un-pol light sources. The inset shows the direction of the E-field in each case. FTIR measurements are plotted in lines and the FDTD simulation results are presented in circles.

The influence of the polarization on the HC LSPR is studied as shown in Figure 7. HC with *L* = 2.0 µm and *g* = 0.1 µm are illuminated under co-pol, cross-pol, and un-pol light sources. Both plasmonic modes, *i.e.* the dipole and multipole, are found insensitive to light polarization. In the simulation, the LSPR positions (black and red circles) of the dipole (at 1630 cm^−1^) and the multipole mode (at 2970 cm^−1^) under co- and cross-pol light are situated at the same frequency. Similarly, the measured LSPR positions (black and red lines) of the dipole (at 1535 cm^−1^) and the multipole mode (at 2755 cm^−1^) under co- and cross-pol light as well as under an un-pol light (green line) are located at the same frequency. Note that the LSPR spectrum under un-pol light slightly exceeds the spectra under a co-pol and a cross-pol. This is due to the fabrication imperfection as shown in the SEM images of Figure 4. Plus, the measured LSPR are red-shifted compared to the simulated ones as previously discussed. The LSPR amplitude is decreased by 30 %. Both spectral results are the consequence of technological imperfection and the inaccuracy of the material parameters between the simulation and the real measurements.

The results of Figure 7 highlight the rotation invariance of the HC antennas so that the incident light polarization will not affect the position of their corresponding LSPR. This feature will be peculiarly useful to improve the signal-to-noise ratio, especially for the subsequently performed sensing experiments, since the totality of the incident light intensity reaches the zone of interest onto the sample without being attenuated by a factor of 1/2 upon navigating through a polarizer.

#### 3.2.3 Local near-field enhancement

Besides the far-field interpretation, the LSPR are accompanied by strong near-field localization and enhancement at the triangle’s pointed extremities forming a hotspot [11]. Indeed, the MIM cavity absorbs the incoming light at the resonance, yielding a large field enhancement because the field gets “squeezed” into the cavity at the edges of the finite antenna, resulting in a lightning-rod effect. By promoting the strong interaction between the oscillating charges on the closely-placed sharp apexes, the near E-field intensity, **|*E*|** ^**2**^ **/|*E***_**0**_ **|** ^**2**^, is accordingly improved inside the activated gap. Such a coupling is forced to occur along the polarization direction. Consequently, under a co-polarized light, the near E-field enhancement and intensity are boosted along the tip-to-tip facing triangles and are affected by this gap value.

**Figure 8:**
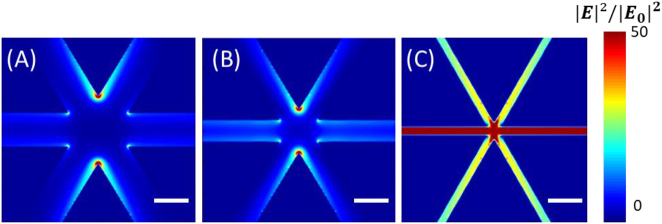
Gap value influence on the local field enhancement and intensity for *L* = 0.8 μm with *g* equal to 0.1, 0.06, and 0.02 µm. Under a co-pol light, the enhanced electric field mapping is collected in the *xy-*plane (A) for *g* = 0.1 µm calculated at *λ* = 3.3 μm, (B) for *g* = 0.06 µm calculated at *λ* = 3.37 µm, (C) for *g* = 0.02 µm calculated at *λ* = 3.5 µm. The color scale ranging from blue to red indicates the minimum to maximum field intensity **|*E*|** ^**2**^ **/|*E***_**0**_ **|** ^**2**^, max (**|*E*|** ^**2**^ **/|*E***_**0**_ **|** ^**2**^) equals 250, 233, 216 while decreasing the *g* value. For better contrast, the maximum color scale is fixed at 50. All scale bars are 0.1 µm long.

In the following, the gap influence on the plasmonic response of the Al-HC is investigated under a co-pol light. The structure’s side length is *L* = 0.8 µm and the gaps *g* are selected to be equal to 0.1, 0.06, and 0.02 µm. In principle, based on our previous work regarding the BT [18], the LSPR red-shifts, and the **|*E*|** ^**2**^ **/|*E***_**0**_ **|** ^**2**^ increases as soon as the value of *g* decreases. To evoke the E-field dependency on the *g* value, a finer mesh override of 2 nm is adapted instead of 5 nm for more accurate E-field mapping. The E-field intensity is collected in the *xy*-plane and represented in Figure 8. Under a co-pol light, the E-field is pinched at the extremities of the tip-to-tip facing triangles.

For *g* = 0.1 µm (Figure 8A), the max **|*E*|** ^**2**^ **/|*E***_**0**_ **|** ^**2**^ equals 250 and appears at 3030.3 cm^−1^ (*λ* = 3.3 µm). It is slightly red-shifted with respect to the far-field LSPR response, *i.e.* reflectance minimum found at 3336.7 cm^−1^ (*λ*_res_ = 2.97 µm). The maximum near E-field is found always at lower energy than the far-field LSPR energy because our plasmonic resonators are conceived as damped harmonic oscillators with natural material losses.

Furthermore, the max value of **|*E*|** ^**2**^ **/|*E***_**0**_ **|** ^**2**^ should increase as far as *g* decreases. However, for a *g* = 0.06 µm, LSPR energetic position appears at 3278.7 cm^−1^ (*λ*_res_ = 3.05 µm), and **|*E*|** ^**2**^ **/|*E***_**0**_ **|** ^**2**^ equal to 233 is found at lower energy, 2967.35 cm^−1^, (*i.e. λ* = 3.37 µm) that is slightly lower than the one found for *g* = 0.1 µm. This result seems illogical until looking at the corresponding E-field mapping in Figure 8B that shows a considerable distribution along the triangle’s neighboring edges. This behavior is emphasized when having *g* = 0.02 µm (Figure 8C). Herein, a max **|*E*|** ^**2**^ **/|*E***_**0**_ **|** ^**2**^ of 216 is found at 2857.15 cm^−1^ (*λ* = 3.5 µm) (red-shifted in respect to the far-field LSPR found at 2906.97 cm^−1^, *i.e. λ*_res_ = 3.44 µm) and is lower than the one found for a larger gap. In fact, for such a small gap, the near E-field is not only enhanced at the sharp tips but also at the corners of the triangles. This is lowering the possibility of the near E-field being largely enhanced by only being pinched at the sharp facing tips. As shown in Figure 8C, this near E-field distribution creates a kind of long-way cavity where the near E-field could be probably delocalized.

In comparison to the Al-BT structure, for *g* = 0.02 µm, **|*E*|** ^**2**^ **/|*E***_**0**_ **|** ^**2**^ of 1250 is found instead of 216, so, in terms of effective E-field enhancement, the BT structure is superior to the HC structure. However, in terms of near E-field enhanced surface, which is vital for molecule-antenna coupling, such a long-way cavity would boost the SEIRA outcome, *e.g.* the enhanced surface in the enhancement factor calculation. In addition, the enhanced near E-field using HC is driven by the light polarization and thus localized at more than 2 tips (surpassing the BT) by turning the polarization direction. Herewith, the enhanced selected-sites will be as well beneficial for sensitive SEIRA sensing.

#### 3.2.4 Arrangement influence on the LSPR

**Figure 9:**
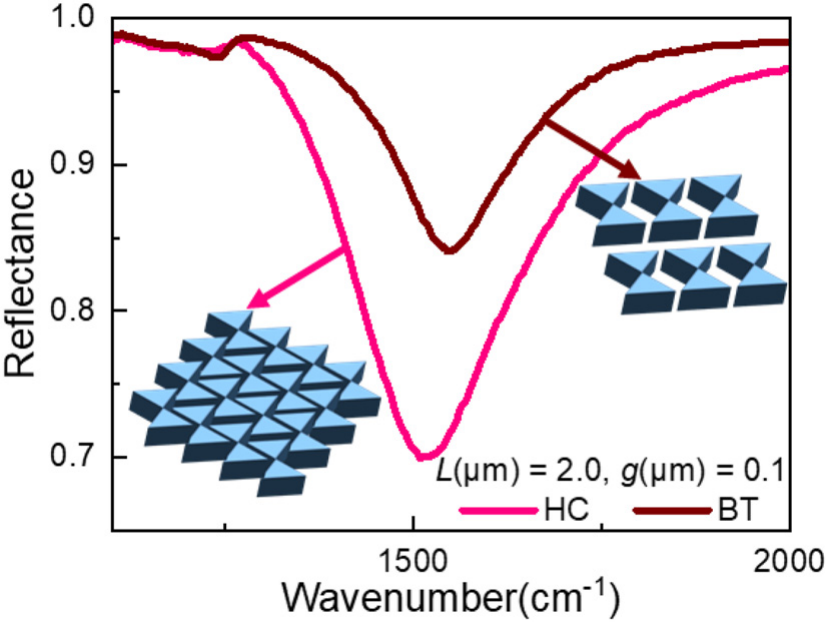
The arrangement influence on the reflectance of both BT (brown curve) and HC (pink curve) nanoantennas (insets), illuminated under a co-pol light source. In both cases, *L* and *g* are respectively fixed at 2.0 µm and 0.1 µm. For a compact arrangement, the LSPR is more pronounced in terms of amplitude and it is slightly red-shifted compared to the one of the BT, as more resonators are interacting.

HC structure might be more interesting than a BT antenna by affording a higher signal-to-noise ratio (SNR). At this stage, a brief comparison between both BT and HC nanoantennas is worth mentioning, as presented in Figure 9. Here, *L* is fixed at 2.0 µm and *g* at 0.1 µm and a co-pol light is applied. For a HC compact arrangement of the triangles (pink curve), one can remark that the amplitude of the LSPR is improved by 20 % compared to BT as simple tip-to-tip triangle arrays (brown curve). This underlines the advantage of the compact nanoantennas organization. Moreover, the compact arrangement is translated into a slight red-shift of the LSPR as the interaction between the adjacent triangles becomes more effective, and thus, arises at lower energy. The LSPR of the HC has shown a slight red-shift (Δ*ω*_res_ ∼ 20 ± 4 cm^−1^) compared to the BT resonance for the same *L* and *g* values. The interpretation of the measured spectra agrees fairly well with the simulation results taking into account the fabrication restrictions.

Plus, the LSPR spectra are large enough, thus, are beneficial for the upcoming sensing application that anticipates a hybrid coupling between the wide *ω*_res_ and narrow *ω*_vib_ frequencies.

## 4 Sensing demonstration

**Figure 10:**
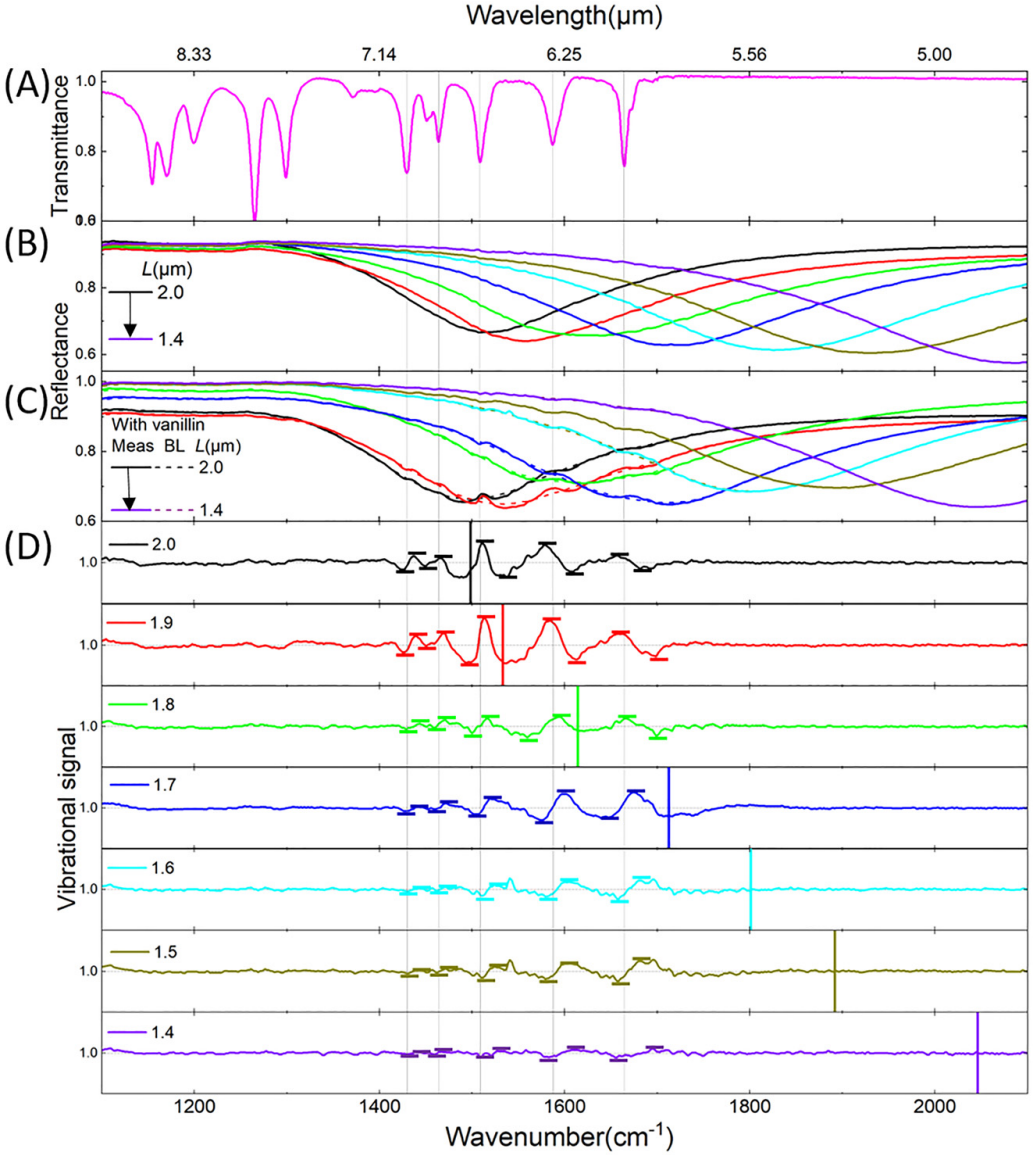
Resonant SEIRA sensing demonstration of vanillin using Al-HC under a co-pol light. The gap value is fixed at 0.1 µm. (A) Transmittance spectrum of vanillin (50 mg/mL) normalized to the one of cleaned KBr platelet. Gray vertical lines eye-guide the IR absorption lines of vanillin. Al-HC of different *L* varied from 2.0 to 1.4 μm with a step of 0.1 μm are covering a wide spectral range from 1400 to 2100 cm^−1^. The corresponding reflectance spectra are acquired before (B) and after (C) the deposition of vanillin. The dashed curves are the corresponding baseline (BL) fitted using Eiler’s smoothing algorithm, excluding the vibrational features (Fano-profiles). (D) Vibrational signals are normalized by the BL for each HC array (side length *L* given in microns). The plasmonic resonance positions *ω*_res_ are marked with thick-colored vertical lines. The signal strengths of the enhanced molecular vibrational modes are obtained as a peak-to-peak value defined by the horizontal ticks.

The SEIRA investigation is first presented from a far-field perspective in Figure 10. As a starting point, the vanillin absorption lines from 1100 to 2100 cm^−1^ are obtained from a transmittance measurement of a highly-concentrated vanillin solution (50 mg/mL) in IPA, deposited on an IR-transparent KBr crystal, as revealed in Figure 10A.

To evaluate the features of the SEIRA spectroscopy using HC nanoantennas, a 2 mg/mL solution of vanillin/IPA is prepared. A 1 µL droplet is then released onto the HC sample surface. Around 10 nmol are hence dropped on the whole transducer surface (having the vanillin with a molecular mass of 152.1 g/mol). The distribution of vanillin molecules is assumed to be homogenous on the 1 cm^2^ sample surface. Indeed, the adsorption of vanillin on Al follows a Langmuir isotherm model where the adsorbed molecules do not interact and mono-layer coverage could be assumed once the vanillin is dropped onto the surface [32]. So, 0.2 ng of vanillin are located within the zone of 100 × 100 μm^2^.

The SEIRA behavior using the HC nanoantennas is valued once far-field measurements are done using the single-element detector under a 36 × IR objective (NA = 0.5). The reflectance area of the optical transducer is selected by a manual knife-edge aperture delimiting the 100 × 100 μm^2^ tailored zone full of HC patterns. Figure 10 presents the achieved reflectance measurements under a co-pol light, on the same sample position without (Figure 10B) and in presence of (Figure 10C) the vanillin/IPA (2 mg/mL) droplet. The coupling between the wide *ω*_res_ and narrow vibrational modes *ω*_vib_ frequencies is studied through the obtained vibrational signals (Figure 10D), known as asymmetric Fano-like profiles. Five narrow IR absorption lines of the vanillin for wavenumbers higher than 1400 cm^−1^ are interfering within broad *ω*_res_ corresponding to Al-HC arrangement with *L* varying from 2.0 to 1.4 µm with a step of 0.1 µm. The five absorption features of vanillin are situated at 1429.8, 1464.1, 1508.9, 1587.5, and 1664.6 cm^−1^ (following the vertical gray lines along Figure 10). These lines correspond closely to C=O, CH, CC, CO, and CH_3_ stretching, in-plane bending, and asymmetric deformation vibrational lines [33]. Note, the interaction of the molecules with the substrate and their medium (solid and liquid) is directly affecting their binding energy. In line with this, the experimentally found vibrational lines are slightly shifted compared with the analytical ones.

In Figure 10D, the vibrational signals are calculated thanks to the Eiler’s least square smoothing algorithm [34], by normalizing the reflectance curve for each HC array to their corresponding baseline (BL, dashed lines in Figure 10C). The efficiency of the occurring coupling is interpreted through the strength (peak-to-peak value) and the shape of the Fano-like vibrational signals and is modulated by a tuning ratio *ω*_vib_/*ω*_res_. The vibrational signal strength tends to be maximized for the different *L* values once the tuning ratio *ω*_vib_/*ω*_res_ is around 1 (typically around the colored vertical lines) as shown in Figure 10D. The maximum amplitude of the vibrational signal states the best spectral overlap between *ω*_res_ and *ω*_vib_. Plus, the tuning ratio also affects the shape of the vibrational signals. For example, selecting *ω*_vib_ = 1664 cm^−1^, for coupling tunings above 1 (Figure 10D red curve), the line shape keeps on its asymmetric silhouette but reverses its minimum and maximum compared to the tunings below 1 (Figure 10D blue curve) [9]. It is crucial to mention that similar results are found under un-pol light.

To evaluate the SEIRA sensitivity of the Al-HC arrays, the enhancement factor, *EF*, for the five IR lines of the vanillin were examined according to the spectral tuning ratio. Equation (5) defines EF as the following:

(5) $$EF=\left(S_{\text{SEIRA}}/S_{0}\right)\times \left(A_{0}/A_{\text{SEIRA}}\right)$$

where *A*_SEIRA_ and *A*_0_ denote, respectively, the areas covered with molecules in SEIRA and the reference measurements. *S*_SEIRA_ and *S*_0_ are, respectively, the enhanced and unenhanced signal strengths. From the measurements on a metallic surface, the unenhanced signal strengths, *S*_0_ are found in the order of 2 − 5 × 10^−3^ for the absorption lines. The signal strengths, *S*_SEIRA_, are in the order of 2.5 × 10^−2^, as measured in Figure 10. This value is slightly higher than the retrieved values for Al-BT under a co-pol light (*S*_SEIRA_ in the order of 2 × 10^−2^), and it is related to a signal improvement thanks to compacting the nanoantennas into the characterized zone. Under un-pol light, *S*_SEIRA_ are in the order of 4 × 10^−2^, two times greater than the *S*_SEIRA_ values measured under a co-pol light, and it is due to the improvement of the SNR once the totality of the incident illumination intensity reaches the zone of interest onto the sample without being attenuated.

*A*_0_ is defined as the repetitive surface entity (unit cell) and is analytically calculated as developed in Equation (6):

(6) $$A_{0}=3\times \sqrt{3}/2\times a^{2};\;a=L+\sqrt{3}\times g$$

**Figure 11:**
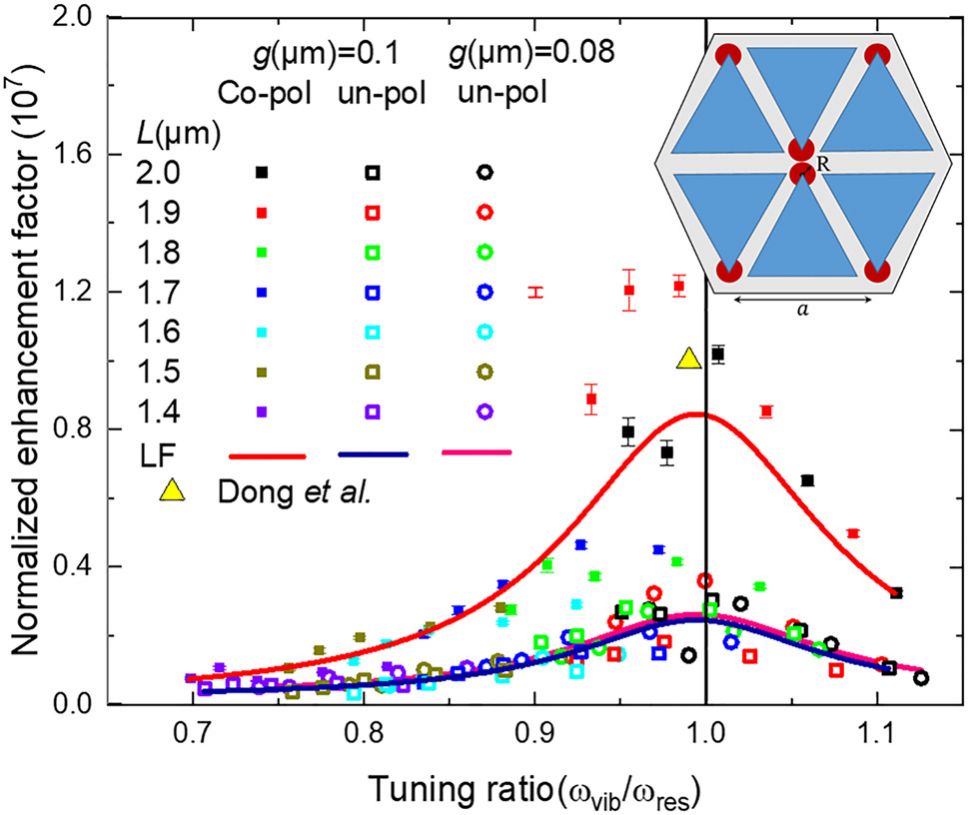
Overview of the normalized enhancement factor (*EF*) of the five absorption features of vanillin versus the tuning ratio *ω*_vib_/*ω*_res_. Under a co-pol light, the *EF* are studied for HC with a *g* = 0.1 µm. The inset shows both *A*_0_ and *A*_SEIRA_ under a co-pol light. Note, under un-pol light all the triangles tips will be activated. The vibrational signal enhancement is generally maximized for a tuning ratio below 1 (black line) as found with a Lorentz fit (LF), declaring the perfect overlap between vibrational modes and plasmonic resonances. The thick error bar represents the systematic error related to *A*_SEIRA_ estimation, to be introduced to the maximum *EF* value. Additional *EF* sequences are presented for HC with *g* = 0.1 and 0.08 µm illuminated under un-pol light. The maximum *EF* is reduced by a factor of 3 under un-pol light which is inversely proportional to the *A*_SEIRA_ prompted by the light polarization.

The total antenna area, *A*_0_, yields 0.2 pg per HC antenna, the half of the mass that is covering the *A*_0_ for BT, as the characterized surface of HC is half of the BT’s one (*S*_objective,HC_ = 100 × 100 µm^2^ < *S*_objective,BT_ = 230 × 90 µm^2^). For an entirely covered antenna, 7 × 10^8^ molecules per antenna (lower than 15 × 10^8^ molecules per BT antenna) are estimated. However, the probed molecules are outlined in the antenna intense near-field hotspots triggered by the light polarization (see the inset of Figure 11). Hence, an effective area, *A*_eff_, that is accessible to the analyte is defined as the 5/6 of an 8 nm-radius circular surface centered around the tip, where the local E-field is intensified, excluding thus the internal area of the equilateral triangle tip. Under a co-pol light, *A*_SEIRA_ is counting 6 times the *A*_eff_ *. A*_SEIRA_ represents 0.12 ‰ of the unit cell area *A*_0_ *.* Therefore, approximately 1.3 × 10^4^ molecules are triggered per tip (*A*_eff_). Under un-pol light, the same amount of molecules is retrieved per tip, yet, *A*_SEIRA_ is counting 18 times the *A*_eff_. It is three orders of magnitude lower than the value reported by Barho et al. for vanillin detection (∼5 × 10^7^ molecules per antenna) with highly doped semiconductor periodic rectangular nanoantenna arrays [30]. *A*_SEIRA_ represents 0.37 ‰ of the unit cell area *A*_0_. Despite of the difference in *A*_0_ definition and *A*_SEIRA_ quantification, the retrieved numbers of triggered molecules using Al-HC antennas are in the same order of magnitude as for the Al-BT antennas [18]. It is successfully matching the state-of-art as reported for self-assembled monolayers detection by Halas et al. [22], Neubrech et al. [5], and Brown et al. [14].

Moreover, the normalized enhancement factor *EF* depends on the tuning ratio *ω*_vib_/*ω*_res_ as illustrated in Figure 11 for all the selected *L* values. *EF* is higher than ∼10^7^ under a co-pol light as shown in the *y*-axis of Figure 11. It is identical to the order of magnitude achieved with the Al-BT antennas and it is agreeing with the greatest stated value reported by Dong et al. using 3-nm gapped BT antennas onto an optical cavity [15]. The *EF* maximum value of 1.2 × 10^7^ is found for a tuning ratio *ω*_vib_/*ω*_res_ = 0.98. It is achieved for the absorption line *ω*_vib_ situated at 1587 cm^−1^ with an Al-HC array of *L* = 1.9 μm resonating at a *ω*_vib_ = 1533 cm^−1^. It is four orders of magnitude higher than the reported value by Vogt et al. for IR sensing (*EF* ∼ 10^3^ using Au nanorods) [9].

Under un-pol light, the maximum *EF* is established around 3 × 10^6^ for *g* = 0.1 µm (black square in Figure 11) and around 3.6 × 10^6^ for *g* = 0.08 µm (red circle in Figure 11). It is reduced by a factor of 3 compared to the previously mentioned *EF*. From a near-field point of view, this result seems to correlate with Equation (5) as *EF* is inversely proportional to *A*_SEIRA_. Hence, the maximum *EF* is reduced under un-pol light (triggering 18 tips) versus the one retrieved under a co-pol light (triggering 6 tips). Moreover, despite of the SNR improvement by a factor of 2 when removing the polarizer, the anticipated results under a cross-pol light should be taken into consideration as the un-pol light is embracing both polarization cases. Here, the hypothesis reclaims a drop-off in the *EF* values under cross-pol light that is translated into a total defeat of those under un-pol light. Based on a simulation under cross-pol, the near E-field intensity, where the SEIRA mainly arises, is about half of the intensity value that is triggered under a co-pol light. This near-field result supports the previous hypothesis and justifies the *EF* slight dropping under an un-pol light.

## 5 Conclusion and perspective

In this work, a multiresonant optical transducer of Al-HC is integrated within a MIM configuration. The nanonantennas are tailored by combining a novel patterning tactic using electron-beam lithography and a challenging dry etching. The dependency of the nanoantenna’s resonance (*ω*_res_) on their geometric parameters, *e.g.* the size and the gap spacing, is studied in detail. A linear dependency is found between *ω*_res_ and 1/*L*. By considering the Fabry–Perot phase matching condition, a reflection phase *ϕ*_*r*_ around $\frac{\pi }{3}$ is found for the fundamental mode of our MIM system. By varying the size of the HC nanoantennas, a wide IR spectral range is successfully covered. The far-field optical response of Al-HC has been numerically modeled and experimentally verified. Good agreement was found between both approaches. Thanks to the symmetry invariance of this hexagonal arrangement, its corresponding resonance is proved to be insensitive to the light polarization, leading thus to an improvement of the SNR by a factor of 2 while removing the light polarizer. Nevertheless, the near-field localization will be driven by the light polarization. Accordingly, we can conceive a plasmon-mediated and site-selective immobilization of biomolecules (DNA, proteins, amino acids) [35] and nanoparticles (quantum dots) [36]. Besides this appealing feature, the HC arrangement, due to its compactness, surpasses the BT arrays by exhibiting an extra increase of the SNR ratio. To conclude, HC nanoantennas are well-adapted for an accurate vibrational SEIRA sensing demonstration. Indeed, the far-field and near-field results are matching the SEIRA state-of-art. In addition, the arrangement of the antennas boosts their reflectance amplitude, and in consequence, the amplitude of the vibrational signals *S*_SEIRA_. Under a co-pol light, an enhancement factor, *EF*, higher than 10^7^ is achieved at a tuning ratio below 1 and is perfectly matching the state-of-art of SEIRA using Al-BT. Under un-pol light, despite of the SNR improvement, the *EF* is decreased by a factor of 3 due to the increase of *A*_SEIRA_ (18 triggered hotspots per HC antenna). This valuable activation of *A*_SEIRA_ stimulates an encouraging analysis of a minimal detectable concentration of our analyte. Finally, to improve the SEIRA readout, smaller gaps can be finely designed to offer a greater near-field enhancement inside the gap as shown theoretically. In principle, a stronger E-field intensity and a highly “hotspot-populated” HC unit will impeccably lead to higher SEIRA enhancements. Hence, our propitious Al-HC nanoantennas within a MIM configuration anticipates a mass production of site-selective, sensitive, compact and cost-effective biosensing devices for delicate and broadband IR detection based on smart Silicon technologies.
